# The Effect and Feasibility Study of Transforaminal Percutaneous Endoscopic Lumbar Discectomy Via Superior Border of Inferior Pedicle Approach for Down-Migrated Intracanal Disc Herniations

**DOI:** 10.1097/MD.0000000000002899

**Published:** 2016-03-03

**Authors:** Jinwei Ying, Kelun Huang, Minyu Zhu, Beibei Zhou, Yu Wang, Bi Chen, Honglin Teng

**Affiliations:** From the Department of Spine Surgery (JY, KH, MZ, YW, BC, HT); and Department of Medical Laboratory, The First Affiliated Hospital of Wenzhou Medical University, Wenzhou, Zhejiang, China (BZ).

## Abstract

Transforaminal percutaneous endoscopic lumbar discectomy (PELD) is gradually regarded as an effective alternative to posterior open surgery. However, migrated herniations bring a great technical challenge even for experienced surgeons due to the absence of the appropriate approaching guideline. We aimed to describe a safe and effective approaching technique for the removal of down-migrations on the basis of the clinical outcomes and complications compared with the conventional approaching method.

A total of 45 patients recommended to single-level PELD with foraminoplasty were randomly divided into 2 groups, group A received foraminoplasty via upper border of inferior pedicle, group B was approached through the common transforaminal route. The clinical outcomes were evaluated by Visual Analog Scale (VAS) for leg pain and Oswestry Disability Index (ODI) scores. Then participants were classified into 2 types of migrations (high-grade and low-grade) based on the extent of migration presented on preoperative magnetic resonance imaging (MRI). The various comparisons between the 2 surgical techniques were analyzed.

The postoperative VAS and ODI scores significantly decreased in both of the 2 groups after surgery (*P* < 0.001). The follow-up continued 1 year. With increasing length of follow-up, the disparities in clinical outcomes between the 2 groups were gradually narrowing and there was no significant difference at the end of follow-up (*P* = 0.32; *P* = 0.46). There were no differences in the operation time and duration of hospital stay (*P* = 0.36; *P* = 0.08). The highly migration group in group B showed a significant longer operation time (*P* = 0.02), but the extent of migration did not have a significant influence on the operation time in group A with the modified approach (*P* = 0.19). There were no apparent approach-related complications in group A during the procedure and follow-up period.

Foraminoplastic-PELD via upper border of inferior pedicle can serve as a safe and effective minimally invasive technique for removal of down-migrated herniations. Furthermore, it is essential to identify the radiologic characteristics so as to choose the most appropriate approaching technique.

## INTRODUCTION

Since the introduction of the posterolateral percutaneous nucleotomy by Kambin and Gellman,^1^ the techniques have undergone remarkable evolution. Transforaminal percutaneous endoscopic lumbar discectomy (PELD) has recently been regarded as an alternative technique to classic open discectomy with comparable satisfactory results.^2,3^ The indirect central nucleus decompression of this procedure in the early era has evolved into the direct removal of protruded or extruded fragments.^4–7^ Although the rates of successful outcomes reported are comparable for PELD and microdiscectomy, PELD offers shorter duration of postoperative disability.^2,8,9^

Most current percutaneous discectomy techniques are on the basis of the Kambin transforaminal approach, offering favorable outcomes for soft disc herniation.^6,10^ However, when migrated disc herniations approached by conventional foraminoplasty into epidural space (transforaminal endoscopic spine system [TESSYS] technique), especially the sequestrated ones, it needs to resect partial isthmus and superior articular process.^11^ These may lead to unavoidable risks of facet joints abrasion and incomplete removal of fragments, resulting in segmental instability and subsequent open surgery. Furthermore, the efficacy of the procedure for high-grade migrations was not quite satisfactory, hence open surgery was considered for this type.^12–14^ Indeed, highly migrated herniations are inaccessible through the conventional transforaminal approach because of some major shortcomings such as poor visualization, inadequate exposure, and the inability to reach and grasp herniated fragments.^15^ With the development of endoscopic instruments and techniques, the range of indications for lumbar disc herniation with PELD has expanded.

In this study, we introduce and describe in detail a novel technique to effectively remove these soft down-forward migrated intracanal disc herniations with favorable results and overcome the disadvantages from the conventional approach.

## MATERIALS AND METHODS

### Patient Population and Grouping

From February 2014 to August 2014, 45 consecutive patients in our department were enrolled into our study, who would be treated with posterolateral PELD by an experienced surgeon (Dr Teng). The inclusion criteria were as follows: severe or unbearable unilateral radiating leg pain rather than associated back pain; positive nerve root tension sign like straight leg raising test; and single-level soft down-migrated herniation and corresponding nerve root compression were verified by preoperative magnetic resonance imaging (MRI) and CT. Exclusion criteria were: nonmigrated and up-migrated herniations; segmental instability; bony abnormality, central stenosis, or lateral recess stenosis; disc calcification; and high iliac crest when L5/S1 herniation. Then we allocated these participants into 2 groups by sortition randomization method. As a result, 22 patients were enrolled in group A with the modified approach via upper border of inferior pedicle, 23 patients were enrolled in group B through conventional transforaminal route. All patients blinded to the next procedures. This study was approved by the Institutional Ethical Committee and all of the patients signed informed consent forms for any surgery procedure.

### Clinical Evaluation and Radiologic Examination

The total physical examinations and clinical scores were performed by another orthopedist who did not participate in surgery procedures. The intensity of leg pain was assessed by Visual Analog Scale (VAS) score, clinical function was evaluated on the basis of Oswestry Disability Index (ODI) score. The assessments of VAS and ODI scores were repeated in the preoperative and the postoperative immediate period and also during the entire follow-up process. The time-points of follow-up were scheduled at the end of 1 month, 3 months, 6 months, and 1 year after surgery. Preoperative and postoperative MRI examinations were obtained in all patients routinely to ensure the herniated style and adequate removal.

### Radiologic Classification

**FIGURE 1:**
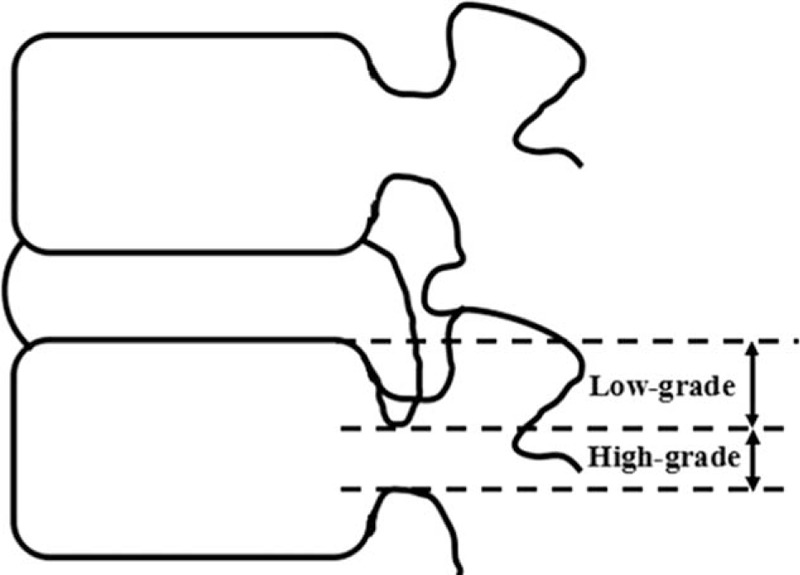
Schematic representation of the extent of the downward migrated herniation in relation to posterior height of disc space.

According to preoperative sagittal MRI, down-migrated herniation was lied away from the extrusion site below the endplate level of the inferior body.^16^ The herniation was called as high-grade migration if the extent of migration exceeded the height of the posterior marginal disc space on the T2-weighted sagittal MRI. On the other hand, it was defined as low-grade migration, whose extent was smaller than the disc space height (Figure 1).^13–16^

### Surgical Technique

Two surgical techniques were performed randomly divided into 2 groups, A and B. The former adopted the transforaminal approach via superior margin of inferior pedicle called as the modified approach, the latter was performed through the classic Kambin transforaminal approach defined as conventional approach. All procedures were performed in lateral decubitus position on a radiolucent table using C-arm fluoroscopy under local anesthesia. Patients could communicated with the surgeon during the entire procedure, which enabled the surgeon to avoid damaging to the neural tissues.

The distance from the skin entry point to midline was determined by preoperative MRI or CT as well as the size of the patient and the dimension of the intervertebral foramen. The skin entry point approximately located 8 to 12 cm from the midline. After the induction of local infiltration anesthesia, an 18-gauge needle was inserted by posterolateral approach under fluoroscopic guidance.

#### The Conventional Transforaminal Approach

The needle tip always aimed at the isthmus of the upper lamina for L4/5, and at the facet joints for L5/S1 and advanced through Kambin triangle between the exiting and traversing nerves (TESSYS technique). When approaching the top of the superior articular process of the inferior vertebra on the anteroposterior view of fluoroscopy, the needle tip should reach the anterior inferior margin of the superior facet on the lateral view. Advancing to the center of spinous processes on the anteroposterior view, the needle tip should close to the posterior vertebral bodyline across the ventral side of the superior facet on the lateral view.

#### The Modified Transforaminal Approach

**FIGURE 2:**
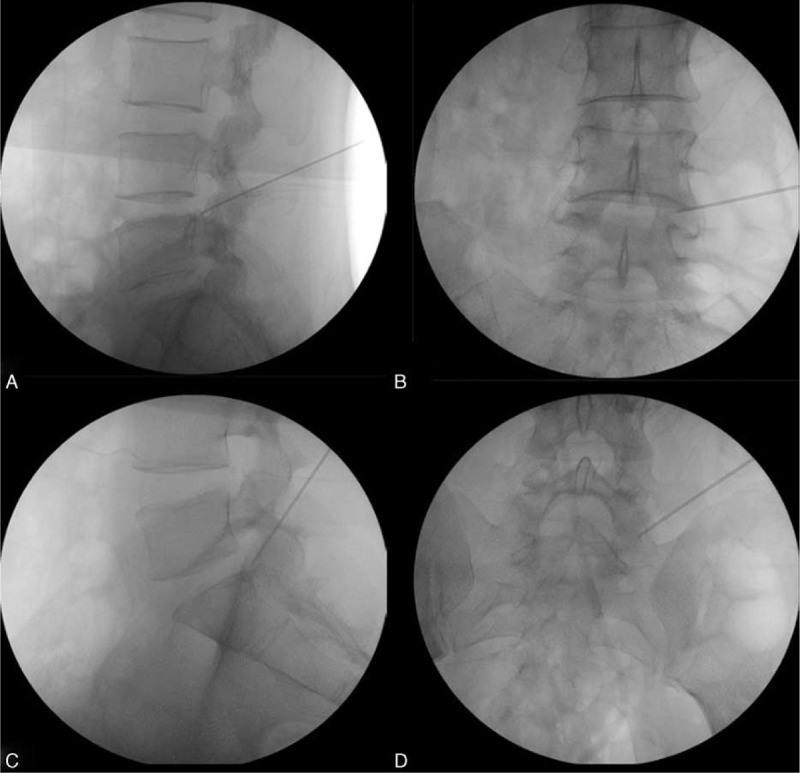
Intraoperative fluoroscopic images showing the modified technique (superior border of inferior pedicle approach), the downward inclination of the needle trajectory with the needle passing through the superior border of inferior pedicle on the lateral radiograph and the needle tip lying at the medial pedicular line on the anteroposterior radiograph (The upper pictures showing the approaching at L4/5 disc level, and the bottom pictures showing that at L5/S1 disc level).

The targeted point was defined as the intersection of one line drawn along the superior margin of the inferior pedicle and the other line drawn along the ventrolateral margin of the superior facet on the lateral view. The skin entry was positioned slightly above the level of the targeted disc with the need tip inclined downward to make an angle of approximately 30° with the endplate (Figure 2). When the needle tip positioned at the midline on the anteroposterior view, it should touch the posterior margin of disc and pass through the superior margin of the inferior pedicle.

The needle was then replaced by a 0.8-mm guide wire with a small stab incision. Sequential reamers were introduced through the guide wire to enlarge the foramen by removal of the partial superior facet when using the conventional approach. In the cases of the modified approach, a partial pediculectomy, the superior and medial border of inferior pedicle, was also needed to widen the foraminal window in order to gain access to the fragment. A bevel-ended, 7.5 mm working cannula was then passed over the obturator with rotation motion. Rotating the cannula and endoscope could offer an all-around visualization. Generally, additional foraminoplastic procedures were necessary in some cases with high-grade migrations. Foraminal ligaments and the bony part of superior facet were removed by laser and the bone cutter under endoscopic view. All these procedures were completed into the epidural space without positioning inside of the disc space.

We also recorded the duration of operation time of all the procedures and postoperative hospital stay in detail.

### Statistical Analysis

The data were statistically analyzed by SPSS 17.0 software. Statistical analysis was performed using independent samples *t*-test and Chi-square test to compare groups for preoperative information. The differences of VAS and ODI between the preoperative and postoperative any time-point in each group were conducted using paired samples *t*-test. The statistical differences in clinical outcome and operation time and hospital stay between the 2 groups were analyzed by independent sample *t*-test. The distinction in the operation time between the high-grade and low-grade migrations was also analyzed by independent sample *t*-test. Statistical significant difference was established at *P* < 0.05 in every analysis.

## RESULTS

### Demographic Data

The baseline demographic and clinical characteristics for each group are summarized in Table 1. There were no statistically significant differences in age, sex, location of herniation, duration of symptoms, the extent of migration, and history of lumbar surgery and trauma (*P* > 0.05).

**TABLE 1:**
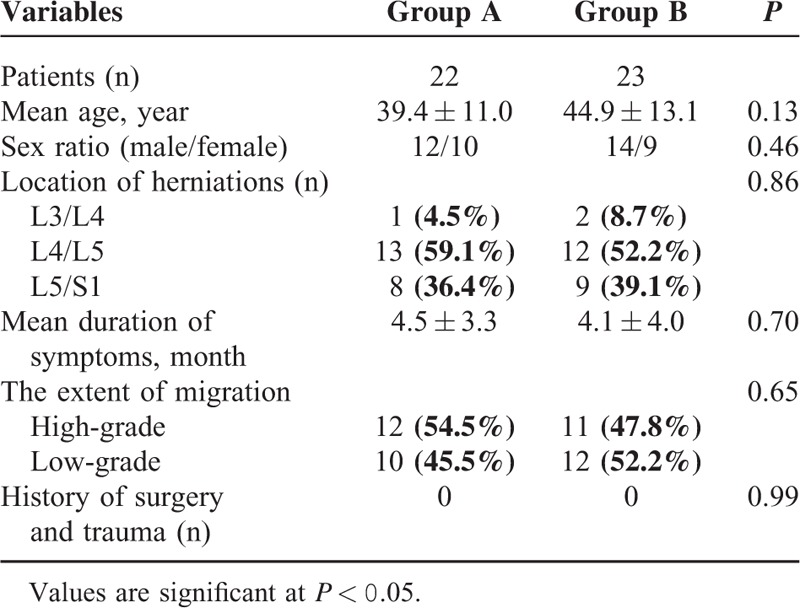
The Demographic and Clinical Characteristics in Each Group

### Preoperative and Postoperative Clinical Assessments

There were no significant differences in the preoperative VAS and ODI scores between the 2 groups (*P* = 0.45; *P* = 0.40). Two patients in group B, because of incomplete decompression and second open surgery, were ruled out follow-up. The remaining 43 patients completed the entire follow-up. The mean preoperative VAS was 5.95 ± 1.33 and 6.30 ± 1.72, which decreased significantly to 1.05 ± 0.90 and 1.30 ± 0.82 at the final follow-up, respectively (*P* < 0.001). And also, the mean ODI score reduced from preoperative 55.44 ± 14.73 and 59.51 ± 16.96 to postoperative 12.42 ± 6.98 and 13.91 ± 6.27 (*P* < 0.001).

**FIGURE 3:**
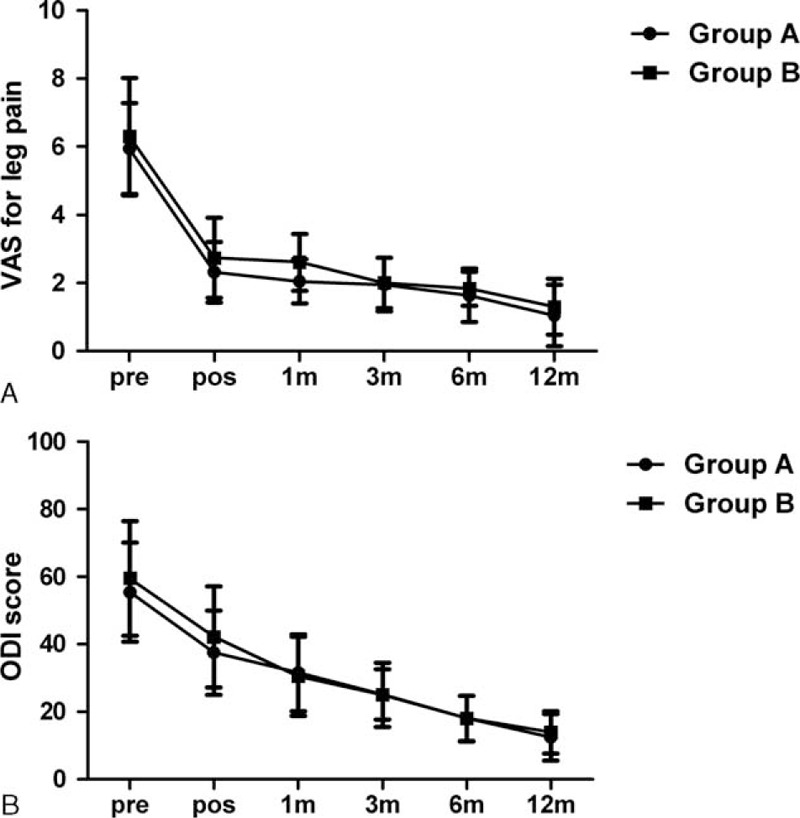
Comparisons of the improvement curves of VAS and ODI scores between the two groups after surgery. The postoperative change tendencies of VAS and ODI scores in the 2 groups are demonstrated in A and B, respectively, pre: preoperation; pos: postoperation; m: month. ODI = Oswestry Disability Index, VAS = Visual Analog Scale.

As can be seen in Figure 3, the postoperative 1-month VAS score in group A was significantly lower than group B (*P* = 0.02). There was, however, no statistically significant difference between the 2 groups in the clinical outcomes at the final follow-up (*P* > 0.05). It was a greater early improvement of VAS in group A, but it tended to equivalence between the 2 groups. On the other hand, the tendency of ODI scores demonstrated 2 significantly indifferent curves for the 2 groups as follow-up time went on (*P* > 0.05).

### Assessments of Hospital Stay and Operation Time

The hospital stay and operation time in each group are demonstrated in Table 2. The duration of hospital stay after surgery was 3.45 ± 1.63 days for group A and 4.57 ± 2.46 days for group B, and so there was no difference between the 2 groups (*P* = 0.08). There was no significant difference between group A and group B in the mean operation time (59.64 ± 11.69 vs 64.00 ± 18.87 minutes, *P* = 0.36). The overall high-grade migration group showed a longer operation time, and this difference had significance (67.91 ± 17.56 vs 55.54 ± 10.73 minutes, *P* = 0.01). And the operation time was significantly longer in those with high-grade migrations in group B (*P* = 0.02), whereas the extent of migration did not have a significant effect on the operation time in group A (*P* = 0.19).

**TABLE 2:**
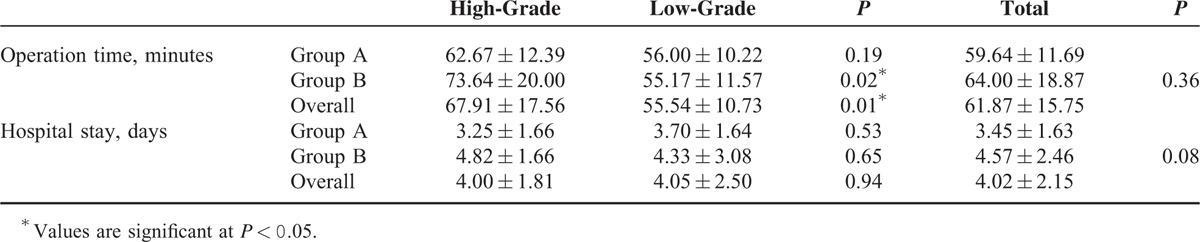
Operation Time and Hospital Stay in Each Group

### Operative Failure and Complications

**FIGURE 4:**
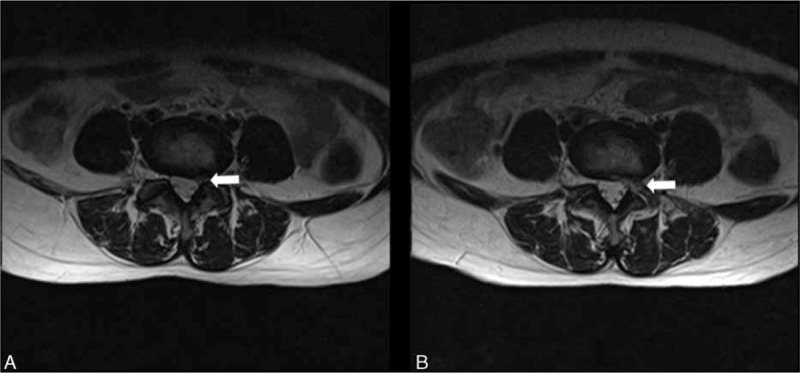
A representative patient with L4/5 left disc herniation who underwent PELD by the conventional foraminoplasty. In picture A, it demonstrates that the extruded nucleus pulposus defects the left L5 nerve root in the epidural space before surgery (white arrow). The postoperative axial MRI findings enable to detect not only the complete removal of disc fragments, but also the damage of articular facet in picture B (white arrow). MRI = magnetic resonance imaging, PELD = percutaneous endoscopic lumbar discectomy.

There were 2 patients in group B and no patient in group A complained of leg pain as before due to incomplete decompression proved by postoperative MRI findings. The herniation style of the 2 patients was classified as high-grade migration. Both of them underwent a subsequent open discectomy. There was no aggravating leg pain in group A because of irritation of neural structures during the puncture procedure. Reversely, there were 5 cases in group B had unpleasant feedback occasionally. As a result, postoperative transient dysesthesia was presented in 3 of the 5 patients, but which was never accompanied at the end of follow-up. The occurrence of postoperative transient dysesthesia was high in group B, especially high-grade migrations (2/3). The evidence of the damage of articular surface in one case from group B could be showed on the postoperative MRI clearly (Figure 4). There were no cases of recurrence, infection, segmental instability, dural tear, or neurologic injury in both groups.

## DISCUSSION

The transforaminal approach characterized by working intradiscal space through the safe triangular working zone,^1^ also known as the “inside-out” Yeung endoscopic spine system technique, is an effective PELD technique for the treatment of protruded and nonmigrated herniations. However, some authors have reported not only a high failure incidence was produced by this technique with indirect decompression because of the incomplete removal of intracanal migrated fragments,^13^ but also the unavoidable nonpathological disc tissue damage might cause unnecessary “postdiscectomy syndrome.”^17–19^ With the development of instruments and techniques, the concept of PELD has changed from intradiscal indirect decompression to epidural targeted fragmentectomy.^15,20^

Several studies reported that removal of the migrations in epidural space with “outside-in” TESSYS technique could be an appropriate approach for treating with intracanal migrated herniations after resection of partial facet joint, however, resulting in a potential risk of neural injury and articular surface damage from reaming.^14,21^ Postoperative dysesthesia is the most common complication, which is caused by approach-related irritation of exiting nerve root.^22^ In addition, incomplete decompression is easy to occur in cases of migrated disc herniation or large central disc herniation.^13,14^

Indeed, disc migration is a very frequent event, and downward migrated fragments are more common than upward ones.^23,24^ This type of herniation is considered by most surgeons to be inaccessible by the conventional foraminal approach due to the inability to grasp fragments, hence open surgery is recommended.^12–14^ When these fragments approached by posterior open discectomy, especially the sequestrated ones, it is inevitable to retract paravertebral muscle, cut lamina and facet joint extensively, which may destabilize the motion segment to aggravate chronic back pain.^25^

In contrast, PELD definitely is advantageous in protecting the normal paraspinal structures, minimizing the potential risks of epidural scarring and segmental instability and selective removal of epidurally extruded disc fragments as well.^2,3,14,15,26^ As reported elsewhere, an optimal trajectory, which means the direction should always aim at the target as close as possible, is the utmost important essential to herniated fragments.^2,6,16^ To achieve access to the epidural migrated fragments, a widening of the foraminal working window is necessary. Ironically, the complications of irritation of neural structures and incomplete removal may be closely related to the approaching procedure in the foraminal area and the inadequate recognition about the radiologic classification of herniations.^6,13,14,27^ Schubert and Hoogland^28^ reported that enlarging the intervertebral foramen near the facet joint to approach migrated discs using special reamers with a high success rate. But the tip of the articular process can be so easily removed by this approach that surface damage increases the risk of the segmental instability.

Regardless of the approaching angle and distance, the exiting nerve root must be protected during the entire procedure. According to the anatomic relationship between exiting nerve root and foramen, a more caudal approach along the superior border of the inferior pedicle is a safe and effective technique to grasp the tail of the fragment and avoid the exiting nerve root successfully. There is a necessary for foraminoplasty to access high-grade migrations. The reasons are as follows: the most frequency of down-migrations presented at lower levels; the gradually decreased intervertebral foraminal diameters from cranial to caudal, especially when degenerative changes resulted from hypertrophy and thickened ligamentum flavum and overriding of facets. Consequently, Choi et al^15^ proposed a foraminoplasty technique to effectively address the soft highly migrated herniations, defined as “enlarging the foramen to reach the ventral epidural space by undercutting the partial ventrolateral area of the superior articular process and superior margin of inferior pedicle (no involvement in articular surface) and ablation of the foraminal ligaments, using various sizes of reamers and radio-frequency.” However, the focus of this approach is only to illustrate a high rate of favorable outcomes resulted from the modified foraminoplasty technique. And they did not put emphasis to compare the clinical outcomes and complications with previous conventional technique.

**FIGURE 5:**
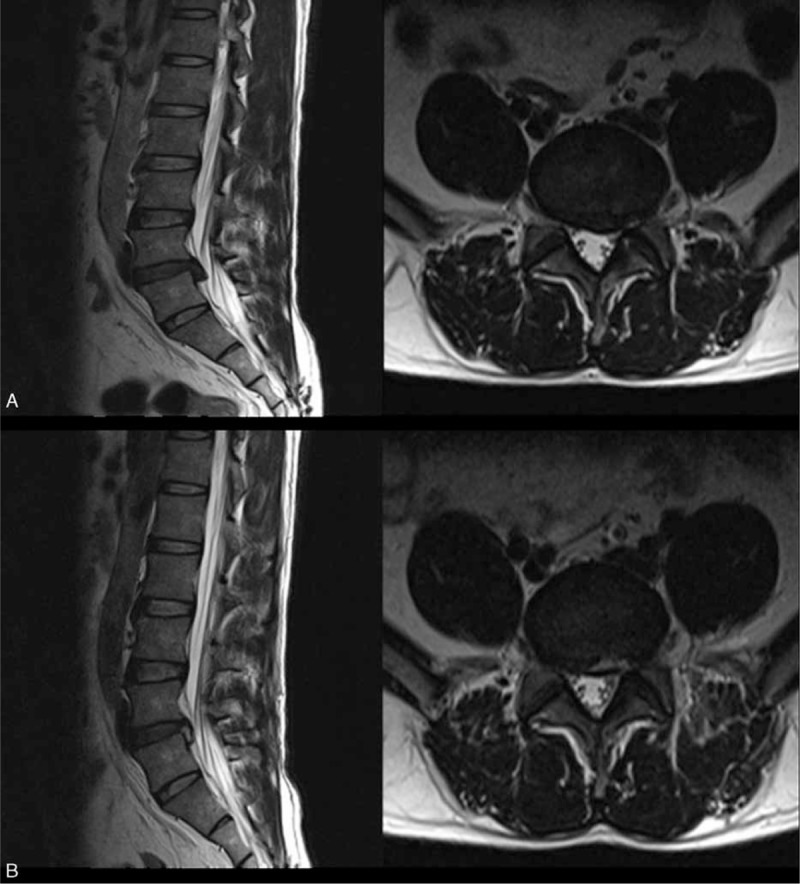
Comparison of magnetic resonance images before (A) and after (B) operation. (A) Preoperative sagittal and axial images showing a highly down-migrated herniation. (B) Postoperative sagittal and axial images of the same patient showing complete removal of the migrated fragments at the 1 month after surgery.

In our study, the authors compared clinical outcomes as well as some complications in down-migrated discs treated with the modified approach and the conventional approach. Although the improvement of VAS score with the modified approach was greater in the early follow-up period, they gradually tended to equalize during the follow-up. It is revealed of a possibility of a gradual self-repair process of the neurologic function or the partial patients in group B accepted the second open revision surgery and achieved good curative effect subsequently. Furthermore, the patients with modified approach not only rarely felt aggravating radicular pain due to the neural stimulation by instruments during the approaching procedure, but also barely suffered from the postoperative dysesthesia. These results may indicate that removal of the migrated fragments is operated more thoroughly with few irritation of exiting nerve root using the upper margin of inferior pedicle approach (Figure 5). There was no approach-related complication with the new technique even during the entire follow-up period. Therefore, we believe that the PELD with foraminoplasty through the superior border of the inferior pedicle will be a better technique for down-migrated intracanal disc herniations.

There was no significant difference in the operation time between the high-grade and low-grade migrations in group A, which indicates that most of the fragments could be accessible smoothly with the modified approach, regardless of the extent of migration. On the other hand, the significantly longer operation time in group B was observed in the high-grade migration group which demonstrates that surgeons could not remove the fragments easily with conventional approach due to the restriction of the endoscopic view and the unaccessible boundary of the mechanical instruments in that case. In addition, hospital staying between the 2 groups was similar.

There are some limitations in our study. First, we should have required a larger study to improve our statistical correctness. Second, the learning curve of this technique is so steep that there is a need to improve the surgeons’ actual operating level. Besides, there were such a few individuals in group B had intraoperative and postoperative complications and accepted open revision surgery because of operative failures known to affect the clinical assessments that it is possible to product some inevitable errors.

Percutaneous endoscopic foraminoplasty via superior border of the inferior pedicle is a safe and effective procedure for down-migrated fragments. There are no significant differences in the eventual surgical effect and operation time compared with the conventional approach. However, it has advantages in the treatment of high-grade migrations and avoiding invading articular surface as much as possible to maintain stable in future and reducing the probability of dysesthesia because of irritation to exiting nerve root.
